# Distinct bacterial community structures with abundant carbon degradation and sulfur metabolisms found in different sea-ice types from the Central Arctic Ocean

**DOI:** 10.1128/spectrum.01291-25

**Published:** 2025-10-08

**Authors:** Siddarthan Venkatachalam, Mats A. Granskog, Rafael Gonçalves‐Araujo, Dmitry V. Divine, Puthiya Veettil Vipindas, Thajudeen Jabir, Ahammed Shereef, Anand Jain

**Affiliations:** 1 Arctic Ecology and Biogeochemistry Section, National Centre for Polar and Ocean Research, Ministry of Earth Sciences (Govt. of India), Vasco-da-Gama, Goa, India; 2 Norwegian Polar Institute, Fram Centre, Tromsø, Norway; 3 National Institute for Aquatic Resources, Technical University of Denmark 5205, Lyngby, Denmark; University of Delhi, Delhi, India

**Keywords:** sea-ice microbiome, community structure, genome-resolved metagenomics, metabolic functions, climate change, Arctic Ocean

## Abstract

The rapid decline of sea ice in the relatively understudied Central Arctic Ocean has a significant impact on bacterial biodiversity and the ecological functions they support. We investigated the bacterial community composition and the associated metabolic functions from three geographically distinct sea-ice floes: first-year ice (FYI) at the North Pole and western Nansen Basin and second-year or multi-year ice (SYI/MYI) in the western Amundsen Basin. We resolved the sea-ice bacterial community diversity at species-level precision using a long-read amplicon (*n* = 18) and metagenomic (*n* = 3) sequencing approach. The amplicon sequencing highlighted marked differences in bacterial community structure driven by ice age, floe origin, and environmental factors, demonstrating pronounced vertical structuring among ice horizons. Bacterial taxa like *Paraglaciecola psychrophila, Hydrogenophaga crassostreae, Octadecabacter arcticus, and Polaribacter irgensii* mainly dominated the bottom layers of SYI/MYI, whereas species *Actimicrobium antarcticum, Polaromonas cryoconiti, O. antarcticus,* and *Rhodoferax sp*. dominated the FYI. Similarly, notable taxonomic differences were observed in bacterial taxa inhabiting the surface and interior layers of FYI and SYI/MYI (e.g., *F. frigoris and Hydrogenophaga* sp.). The metagenomic analysis showed the prevalence of sulfur cycling-associated (assimilatory and dissimilatory sulfur metabolism) and complex carbon degradation processes in sea ice. We also elucidated the potential ecological role of novel metagenome-assembled genomes belonging to the genus *Aquiluna* through phylogenomic and pangenomic analyses. Overall, our findings revealed novel insights on the distinct bacterial communities that inhabit ice horizons and their associated ecological functions correlating with sea-ice type, origin, and habitat characteristics in the Central Arctic Ocean.

**IMPORTANCE:**

The Arctic region is warming nearly four times faster than the global average, leading to the continuous replacement of its thick multi-year sea ice with thinner first-year ice. The reduction in Arctic sea-ice cover was previously shown to have cascading effects on sea-ice-associated microbial communities and their role in the functioning of the ecosystem. This study provides the first high-resolution, species-level insight into the bacterial community composition and metabolic potential across different sea-ice types in the Central Arctic Ocean—an understudied yet rapidly changing environment. By combining long-read amplicon and metagenomic sequencing, we uncover distinct bacterial assemblages and functional metabolic roles that were shaped by the ice age and other physicochemical properties. Our findings highlight the ecological importance of sea-ice associated bacterial communities and the prevalence of sulfur metabolism and carbon degradation processes in different sea-ice types found in the central Arctic Ocean through genome-resolved metagenomics.

## INTRODUCTION

Sea ice is considered one of the largest biomes on the planet, covering about 13% of Earth’s surface (1). The extensive sea-ice cover in the polar oceans provides a unique habitat for different groups of microorganisms that participate in various microbially mediated biogeochemical processes (2–5). Sea ice plays a vital role in the distribution and replenishment of nutrients and terrestrial organic matter through their entrainment of riverine inputs during sea-ice formation and transports them across the Arctic Ocean basin (6, 7). However, over the last two decades, the sea-ice extent, thickness, and age have reduced drastically in the Arctic Ocean (8–10). Thick multi-year ice (MYI) has been replaced by thinner seasonal first-year ice (FYI) in many parts of the Central Arctic Ocean (CAO) (8, 11). The reduction in Arctic sea-ice cover was shown to have cascading effects on the entire Arctic marine ecosystem (8, 12).

Sea ice-associated microbial communities significantly contribute to biological productivity and regulate food web structure in the polar oceans (13). The microbial biomass in sea ice is typically dominated by ice algae, which can adapt to very low light conditions (14, 15). These ice algae, along with certain specialized bacterial groups, including Cyanobacteria, are known to produce dimethylsulfoniopropionate (DMSP) in the sea-ice habitat (16, 17). The DMSP functions as both an osmolyte and a cryoprotectant, primarily assisting DMSP-producing organisms in maintaining cellular homeostasis under the high-salinity and freezing temperature conditions found in the sea-ice habitat (18). Additionally, DMSP can benefit surrounding nonproducing heterotrophic microbial groups (e.g., members of the *Rhodobacteraceae* and *Alteromonadaceae*), either directly by stabilizing their osmotic environment or indirectly by serving as a substrate for metabolism through sulfur cycling pathways (5). Earlier sea-ice investigations revealed that prokaryotic communities are compositionally distinct from those in the underlying seawater and are shaped by the extreme environmental conditions of the ice matrix and brine channels, with microbes adapted to high-salinity and subzero conditions (6, 19). The majority of the bacterial populations within sea-ice habitats were reported to be heterotrophs, which play a crucial role in the re-mineralization of available organic matter (20, 21). Several studies have successfully isolated numerically dominant heterotrophic bacterial taxa belonging to Proteobacteria and Bacteroidetes that inhabit the sea ice (17, 22). Similarly, previous studies on different seasons, including winter, spring, and summer, have provided critical knowledge on the bacterial community composition in FYI, MYI, or both in the Arctic Ocean (23–26). These data suggest significant variability in the taxonomic composition of bacterial communities between FYI and MYI, of which FYI generally harbors more compositionally dynamic bacterial community structure in comparison to the more stable communities in MYI. A recent study investigated the bottom cores of land-fast FYI collected in Hudson Bay for deciphering the metabolic potentials of prokaryotic communities associated with sea-ice algae through a metagenomics approach (5). Despite these observations, critical knowledge gaps persist in correlating these taxonomic variabilities with key biogeochemical cycles in different sea-ice types. Similarly, data on bacterial communities that inhabit the ice horizons have been reported only in a few studies (23, 27). Besides, the majority of these studies have been carried out either on land-fast ice or drift ice at relatively lower latitudes of the Arctic and Atlantic Ocean, especially in the Baltic Sea and Fram Strait region (20, 23, 24, 28), while observations in the (for now) perennially ice-covered CAO region are scarce in comparison to other parts of the Arctic Ocean owing to restricted access (26, 29, 30). Similarly, functional metagenomic studies for deciphering the ecological significance of sea-ice associated microorganisms are relatively scarce in the CAO region (29), which limits our understanding of the specific roles these microorganisms play in biogeochemical cycles, ecosystem resilience, and their adaptive mechanisms to extreme polar conditions.

In this study, our aim was to decipher the spatial variability in bacterial communities both horizontally and vertically along with their potential metabolic functions from three geographically distinct sea-ice floes belonging to FYI/SYI/MYI in the CAO. We have used long-read sequencing technology from Oxford Nanopore (31, 32) to obtain species-level taxonomic resolution of sea-ice associated bacterial communities and to assess their metabolic functional capabilities in Arctic sea ice. As climate change accelerates the loss of MYI and its replacement by more transient FYI in the CAO, the resulting increase in community variability may lead to altered nutrient dynamics and have a potential impact on biogeochemical cycling. Considering the different oceanographic settings across the Arctic Ocean, we hypothesized that bacterial community compositions and their distribution patterns could reflect a combination of environmental and physicochemical factors such as sea-ice origin and age, and that microbially mediated metabolic processes (carbon, nitrogen, sulfur cycles, etc) associated with these communities would also be affected in the CAO.

## RESULTS

### Environmental, physicochemical settings, and inference of the age of sea ice

**Fig 1:**
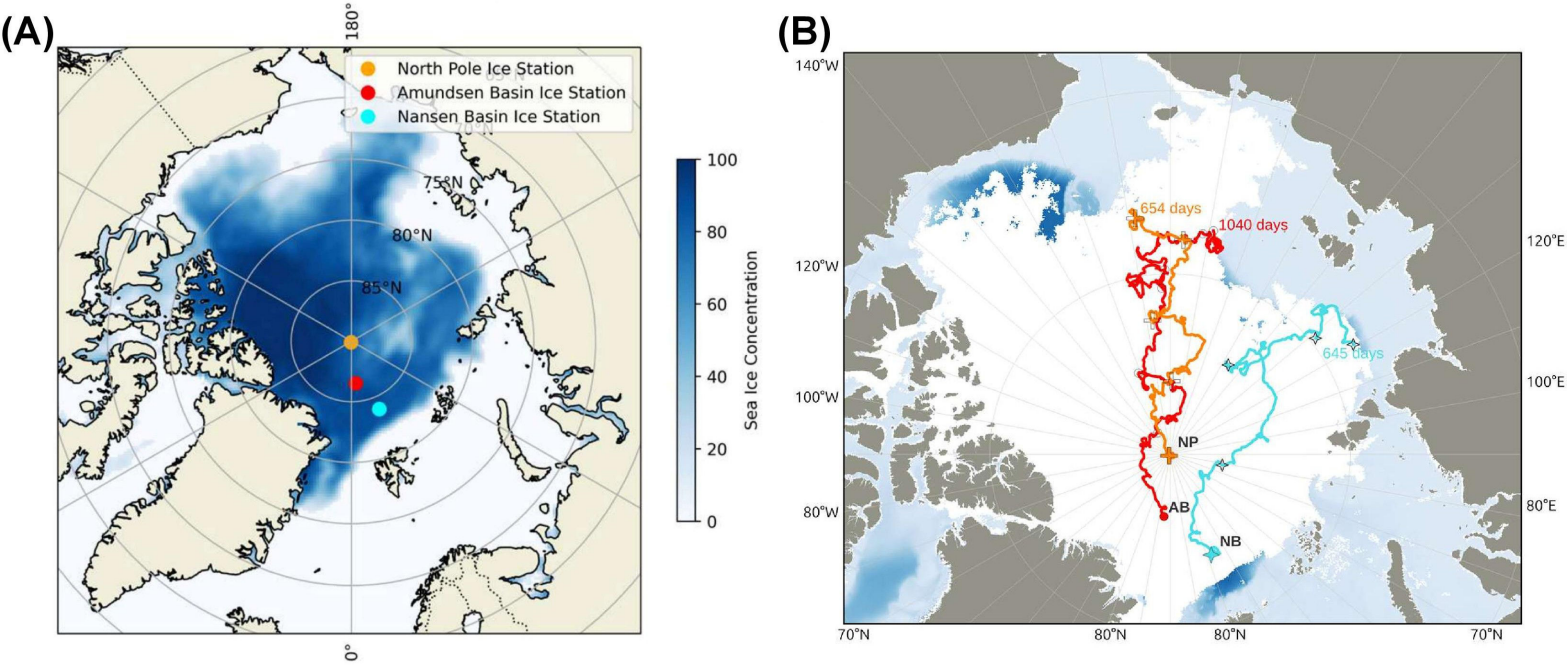
(**A**) Monthly mean sea-ice concentration in August 2022 provided by ECMWF ERA5 (https://cds.climate.copernicus.eu/datasets/reanalysis-era5-single-levels-monthly-means?tab=overview) showing the locations of three ice stations (33). (**B**) Sea-ice back-trajectories were calculated using data from the EUMETSAT Ocean and Sea Ice Satellite Application Facility (EUMETSAT OSI SAF OSI-450-a1, https://osi-saf.eumetsat.int/) and give an indication of the oldest ice that is possible to find in the region where the ice floes were sampled (34). NP: North Pole (yellow); AB: Amundsen Basin (red); NB: Nansen Basin (cyan); back-trajectories. The number of days for each back-trajectory is also indicated.

**Fig 2:**
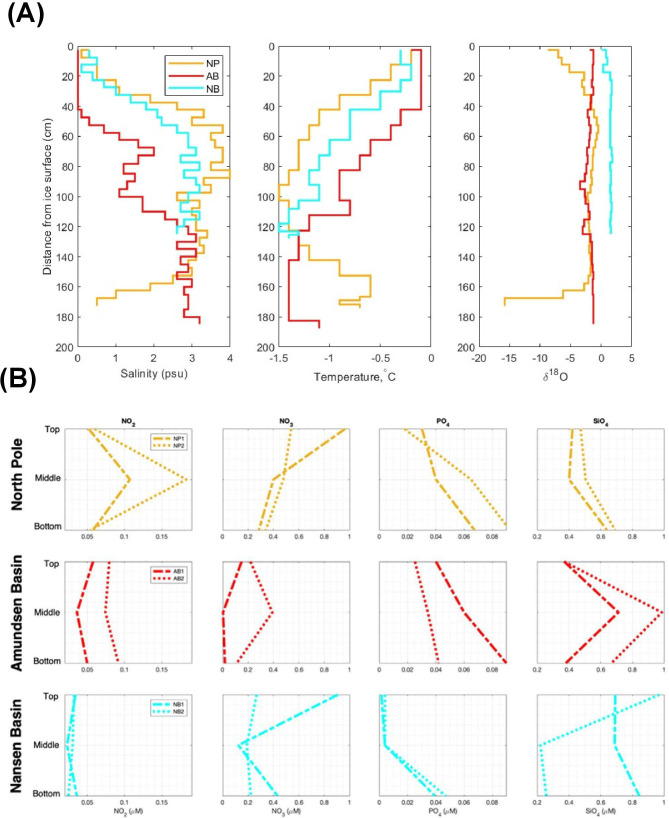
Physicochemical properties of sea-ice cores collected from the CAO region. (**A**) Profiles of salinity, temperature, and δ^18^O (‰). (**B**) Dissolved nutrient concentrations (µM): NO_2_, NO_3_, PO_4_, and SiO_4_ of the sea-ice cores (top, middle, and bottom sections) collected from the CAO region: North Pole (NP; top panel), Amundsen Basin (AB; middle panel), and Nansen Basin (NB; bottom panel).

The back trajectories suggest that sea ice in the area of the ice stations originated from different regions of the Arctic Ocean (Fig. 1B). The North Pole (NP) back-trajectory ends north of the Chukchi Sea, while the western Amundsen Basin (AB) back-trajectory ends north of the East Siberian Sea, and for western Nansen Basin (NB), it ends in the northern Laptev Sea. The back-trajectory analysis revealed that the oldest ice at the NP and NB could be about 2 years old and 3 years old, respectively. At the NP ice station, floe-level ice thickness varied from 1.5 to 2.5 m with an average of 1.9 m, while at the coring site, the ice was about 1.8 m thick (Fig. S1A). At the AB station, floe-level ice thickness ranged from 1.3 to 2.0 m with an average thickness of 1.6 m, while at the coring site, it was about 1.8–1.9 m thick (Fig. S1B). At the NB station, level ice thickness ranged from 1.2 to 2.5 m with an average of 1.6 m, while the coring site was about 1.3 m thick (Fig. S1C). The salinity profiles at every ice station showed low salinities at the surface and increasing with depth, typical for summer melt. The lower parts of the NP and NB stations had a relatively high salinity compared to that at the AB station (Fig. 2A; Table S1). Similar to salinity, the temperature profile (0°C to −1.5°C) was also found to vary only slightly between the three ice stations (Fig. 1B).

The NP ice station had negative oxygen isotope signals at the very top and very bottom of the sea-ice core indicative of contributions of meteoric water (35). For the NB sea-ice core, the δ^18^O profile was very distinct in comparison to the other two ice stations, with much higher values (Fig. 2A). Among the nitrogen-derived compounds, NO_3_ presented the highest concentrations ranging from 0.003 to 0.96 µM, whereas NO_2_ concentrations ranged from 0.02 to 0.18 µM. PO_4_ and SiO_4_ ranged from 0.001 to 0.09 µM and 0.22–0.99 µM, respectively. Overall, the replicates show fairly similar profiles with marked vertical gradients observed in a few cases mainly at the NB ice station (Fig. 2B). Nitrogen levels tend to decrease with depth, and the highest concentrations were at the NP station, whereas the lowest were observed in the AB. PO_4_ levels increased with depth with the NB cores presenting lower concentrations compared to the other two locations. Silicate profiles presented different patterns for each of the sampling areas, showing overall agreement among the replicates, except for NB.

### Bacterial community composition and species-level taxonomic classification using long-read amplicon sequencing

**Fig 3:**
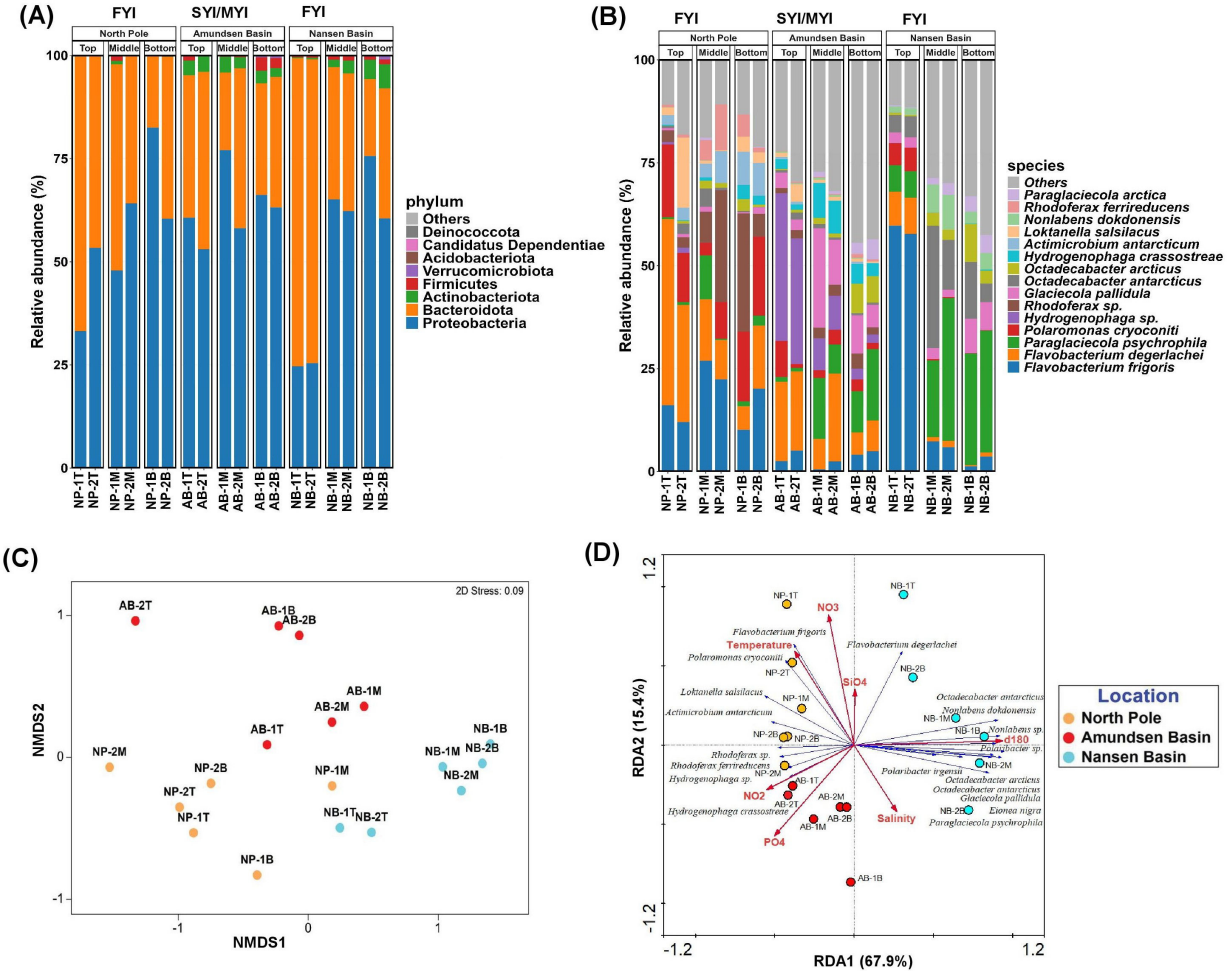
Sea-ice bacterial community structure and biogeographical distribution patterns in the samples collected from the CAO region. Taxonomic classification of sequence reads showing (**A**) the relative abundance at the phylum level and (**B**) the top 15 dominant bacterial species identified from the sea-ice core samples. (**C**) Biogeographical distribution of bacterial communities among three sea-ice stations based on non-metric multidimensional scaling plot (NMDS) analysis. (**D**) Influence of physicochemical factors on the identified dominant bacterial genera from the three CAO ice stations. The red arrows represent measured environmental variables, and the blue arrows represent dominant bacterial genera. The sea-ice core samples from three different ice stations were labeled with unique color codes.

The amplicon sequencing run yielded about a total of 3 million good-quality reads after quality screening (Qscore >7) and adapter trimming among the analyzed samples. A summary of sequence reads obtained from each sample is given in Table S2. The taxonomic classification revealed that sea-ice core samples comprise nine bacterial phyla, of which the abundance of dominant phyla differed among three ice stations and ice horizons (Fig. 3A; Table S3). For example, Proteobacteria were often predominant in bottom ice cores at NP (60%–83%), AB (63%–66%), and NB (60%–75%) ice stations. The Bacteroidota phylum was found to be more dominant in the top cores of NP (46%–66%) and NB (74%) ice stations. The Actinobacteriota phylum, representing up to 6% of total reads, was mainly identified in the sea-ice cores collected from AB and NB ice station samples. The study also features the accurate identification of 503 bacterial taxa up to the species (Table S3), of which 146 bacterial species were commonly shared among all three ice stations (Fig. S2). Several bacterial species were found unique across each ice station with different relative abundance profiles. Among them, NB had a higher number of unique species (*n* = 206) in comparison to NP (*n* = 24) and AB (*n* = 29) ice stations. Of the ice horizon, the bottom core had more unique species (*n* = 63) than the top (*n* = 22) and middle cores (*n* = 26). Based on Lefse analysis, several statistically significant, differentially abundant bacterial taxa (*n* = 132) among each ice station, ice horizon, and ice floe age have also been identified and illustrated in the supplementary information (Fig. S2; Table S4). Among the NP ice station, *Flavobacterium degerlachei* (6%–45%), *F. frigoris* (10%–23%), and *Polaromonas cryoconiti* (up to 19%) were identified as dominant bacterial species across all ice horizons (top, middle, and bottom), whereas *Rhodoferax* sp. Gr-4 (up to 28%), *R. ferrireducens* (up to 11%), and *Actimicrobium antarcticum* (up to 8%) were mainly found abundant in the middle and bottom cores (Fig. 3B). Among the AB ice stations, marked differences were observed in the abundance of the community composition between ice horizons. For example, *Hydrogenophaga* sp. PAMC20947 (up to 35%) and *F. degerlachei* (up to 20%) were found to be the dominant taxa inhabiting the top cores, while *Glaciecola pallidula* (up to 24%), *Paraglaciecola psychrophila* (up to 15%), and *H. crassostreae* (up to 8.5%) were found to inhabit the middle cores. Similarly, bottom cores were mainly dominated by *P. psychrophila (up to 17%*), *Octadecabacter arcticus* (up to 8%), and *Polaribacter irgensii* (up to 3%). At the NB ice station, top cores were mainly inhabited by *F. frigoris* (up to 60%) and *F. degerlachei* (9%), while middle and bottom cores were dominated by *P. psychrophila* (up to 35%), *Octadecabacter antarcticus* (up to 30%), and *Nonlabens dokdonensis* (8.5%). Overall, bacterial community composition was found to have marked differences between different ice stations and ice horizons.

### Biogeographical distribution patterns and influence of physicochemical factors on sea-ice bacterial communities

The non-metric multidimensional scaling (NMDS) further revealed that the bacterial community structure of sea-ice cores collected across three ice stations was significantly different (Fig. 3C; Table S5). The redundancy analysis (RDA) was used to study the influence of various physicochemical properties of the sea ice in determining the bacterial community composition (Fig. 3D). The axes (RDA1&2) accounted for about ~83% of the total variance observed in the relationship between species composition and various environmental factors tested (Fig. 3D). For NP, this showed strong correlation with environmental factors such as temperature, NO_3_, and NO_2,_ which had more influence on the dominant bacterial species like *F. frigoris, P. cryoconite, Loktanella salsilacus, Actimicrobium antarcticum, Rhodoferax* sp., *R. ferrireducens,* and *Hydrogenophaga crassostreae*. At AB, PO_4_ showed a correlation with *Hydrogenophaga crassostreae*. Similarly, at the NB station, salinity, oxygen isotope, and SiO_4_ showed a correlation with *F. degerlachei, Paraglaciecola psychrophila, Polaribacter sp., Polaribacter irgensii, N. dokdonensis, O. arcticus, O. antarcticus, and Eionea nigra*.

### Reconstruction of MAGs through genome-resolved metagenomics

**Fig 4:**
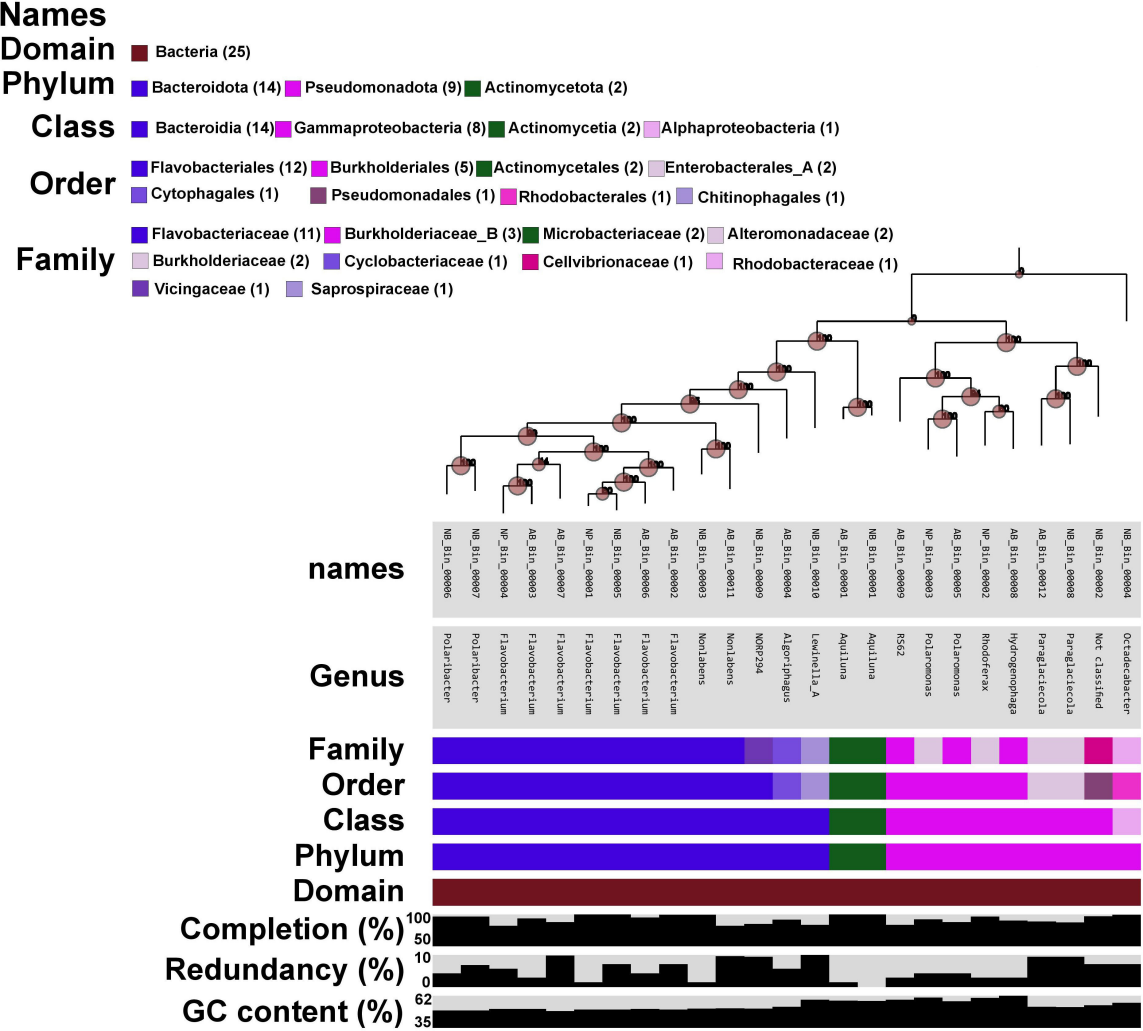
Phylogenomic analysis of all the reconstructed MAGs from the three ice stations in the CAO region. The bar graphs represent the taxonomic descriptions of MAGs at domain, phylum, class, order, family, and genus along with various MAG properties like completion, redundancy, and GC content. The taxonomic description of each MAG at domain, phylum, class, order, and family is color-coded, and their taxonomic detail is provided in the figure (top left). Bootstrap values are represented in the nodes of the tree.

The summary of the sequence reads generated for the metagenome sequencing is given in Table S6. A total of 25 nonredundant MAGs were reconstructed from the metagenomic sequence data. The size of the reconstructed MAGs varied from as low as 1.3 Mb to 7.1 Mb, while GC content among the MAG data sets ranged from 31.7% to 61.7%. The completeness and redundancy of the MAGs varied from ~63%–99% and 0%–9% respectively. The taxonomic classification showed that all the MAGs belonged to 14 different bacterial genera (Table S7) within the phyla of Bacteroidota (*n* = 14), Pseudomonadota (*n* = 9), and Actinomycetota (*n* = 2). The MAGs belonging to the genus *Flavobacterium* were prevalently found among all three sea-ice stations with high abundance and coverage ratio (Fig. S3; Table S8). The study also revealed several genera were found to be unique to sea-ice stations (e.g., NP: *Rhodoferax*; AB: *Algoriphagus, Hydrogenophaga, RS62*; NB: *Octadecabacter, NORP294*). Overall, the abundance and coverage of the reconstructed MAGs were also found to vary among the three ice stations (Fig. S3; Table S8). The phylogenetic diversity among the reconstructed MAGs was diverse in all three sea-ice stations (Fig. 4). The MAGs retrieved within the phylum of Bacteroidota were more diverse, forming four clusters. Similarly, MAGs belonging to the phylum of Pseudomonadota were formed of two clusters. The most prevalently identified *Flavobacterium* genera among all three ice stations were formed of two clusters in the phylogenetic tree, which further proves the high genomic diversity among this group (Fig. 4). The average nucleotide identity (ANI) varied from 64%–100% among the MAGs, suggesting high genomic diversity among the reconstructed MAGs (Table S9).

### Microbially mediated metabolic functions in the sea-ice habitats

**Fig 5:**
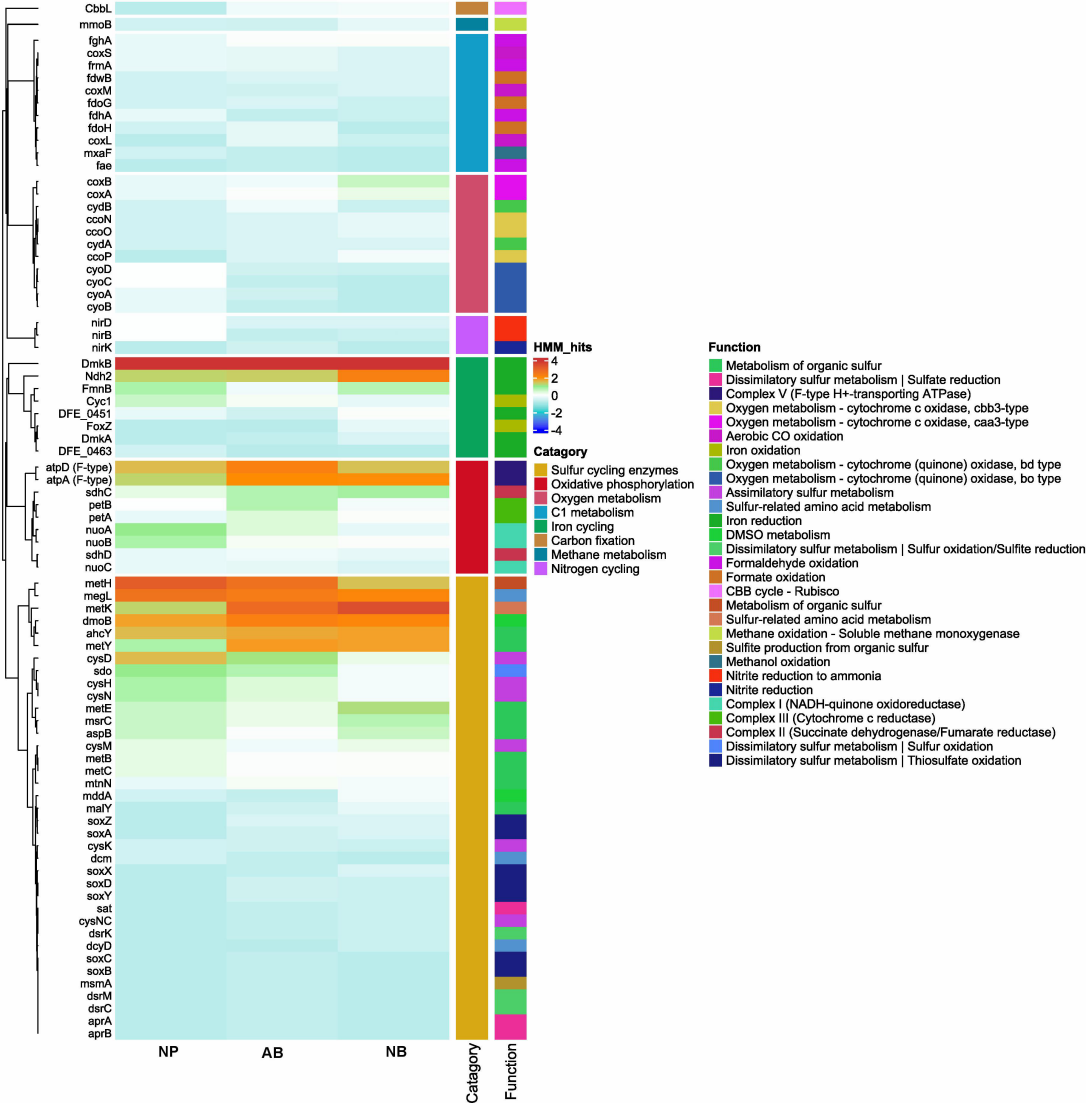
Heatmap showing the various metabolic functions found among the metagenome assemblies at the three ice stations. The metabolic gene counts (HMM_hit) of various processes were normalized using the Z score. A negative z-score (negative HMM_hit) indicates that the abundance of that particular metabolic gene in the sample is below the mean abundance across all samples, while a positive z-score indicates an above-average abundance. The bar plot color codes on the side represent the gene counts associated with various metabolisms and biogeochemical processes found within the metagenome assemblies. The plot was generated using “complexheatmap” using the R package in R version 4.1.3.

**Fig 6:**
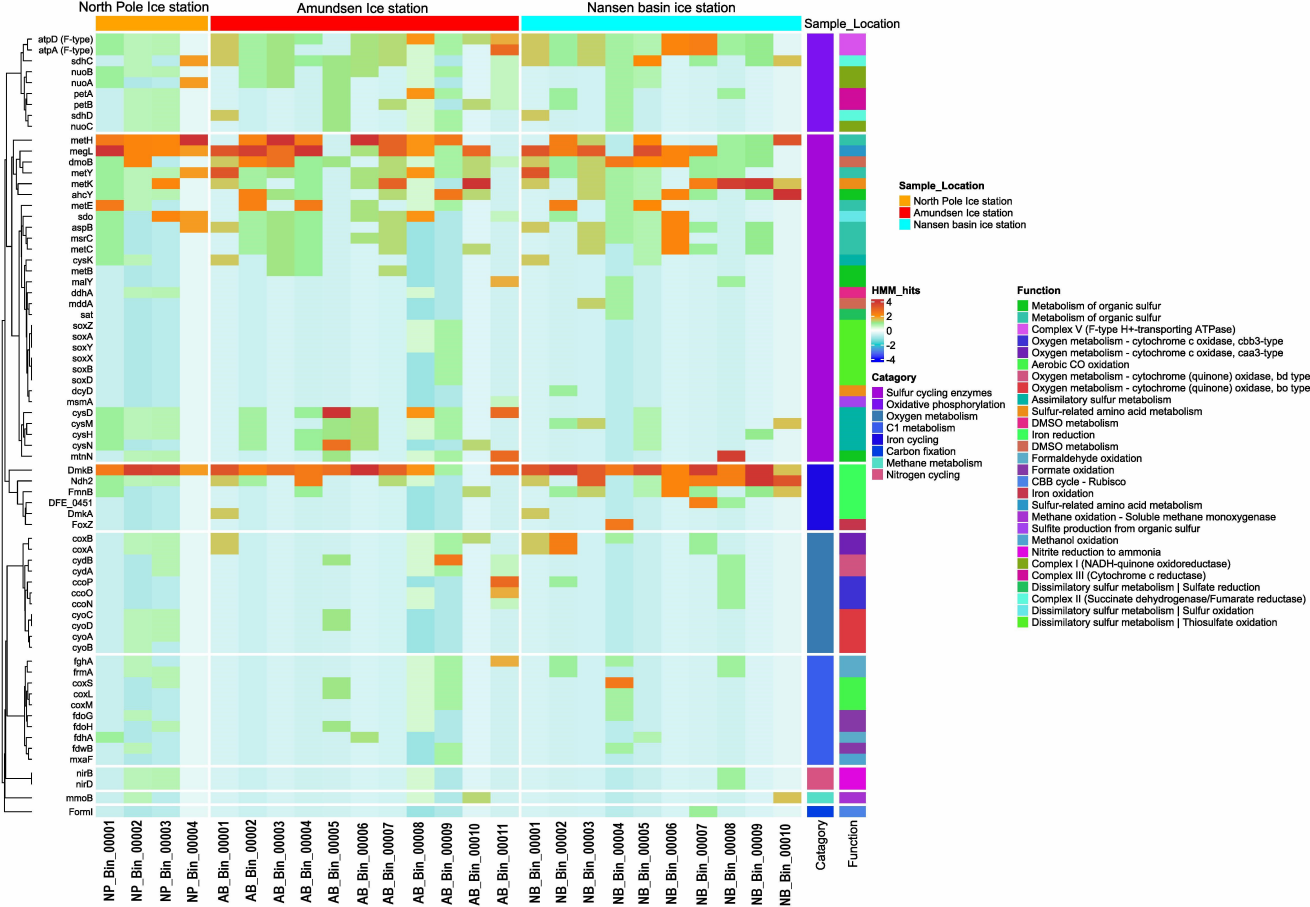
Heatmap showing the various metabolic functions found among the reconstructed MAGs from all three ice stations. The metabolic gene counts (HMM_hit) of various processes were normalized using the Z score. A negative z-score (negative HMM_hit) indicates that the abundance of that particular metabolic gene in the sample is below the mean abundance across all samples, while a positive z-score indicates an above-average abundance. The bar plot color codes on the side represent the gene counts associated with various metabolisms and biogeochemical processes found within the reconstructed MAGs. The plot was generated using “complexheatmap” using the R package in R version 4.1.3.

Metabolic functional analysis was carried out on the metagenome assemblies (Fig. 5; Fig. S4), along with reconstructed MAGs (Fig. 6; Fig. S6) from the samples, to identify the microbially mediated metabolic functions in the sea-ice stations (Table S10). Functional genes encoding for sulfur cycling-associated processes were prevalently identified among all three ice stations (Fig. 5; Table S10), which include assimilatory sulfur metabolism (*cysD, cysN, and cysH*) and dissimilatory sulfur metabolism (*sdo* - sulfur oxidation). These pathways are mainly identified in the MAGs belonging to the members of genera *Flavobacterium, Rhodoferax, Polaromonas, Algoriphagus, Hydrogenophaga, RS62, Nonlabens, Paraglaciecola,* and *NORP294* (Fig. 6; Table S11). However, few functional genes encoded for thiosulfate oxidation (*soxB, soxC, and soxY*), sulfate reduction (*aprA, aprB, and sat*), and carbon fixation process (CBB cycle - Rubisco) were found in the metagenome assemblies of AB and NB ice stations. Bacterial genera *Hydrogenophaga* (AB_Bin_00008) and *RS62* (AB_Bin_00009) were found to encode functional genes associated with thiosulfate oxidation. In contrast, *Octadecabacter* (NB_Bin_00004) and *Polaribacter* (NB_Bin_00007) genera were found to encode for genes associated with sulfate reduction and carbon fixation, respectively (Fig. 6; Table S11). Similarly, complex carbon degradation pathway genes (hexosaminidase and beta-glucosidase), fatty acid degradation (*hadH*), amino acid utilization (*serC and pabC*), iron reduction process (*FmnB, Ndh2, and DmkB*), DMSO metabolism (*dmoB*), and oxidative phosphorylation (*nuoA-C, sdhC, sdhD, petA, petB, etc.*) were also observed prevalently in the AB and NB in comparison to the NP ice station (Fig. S5). These processes were commonly found in most of the reconstructed MAGs. We have also observed a limited number of functional genes associated with denitrification processes (*nirB, nirD, and nirK*) in metagenome assemblies and the MAGs belonging to *Rhodoferax* (NP_Bin_00002), *Polaromonas* (NP_Bin_00003), *Hydrogenophaga* (AB_Bin_00008), and *Paraglaciecola* (NB_Bin_00008). Additionally, some of these MAGs (NP_Bin_00002, AB_Bin_00008, AB_Bin_00011 - *Nonlabens*, and NB_Bin_00010 - *Lewinella_A*) were also found to encode genes related to methane metabolic processes.

### Deciphering the potential metabolic functions of the novel *Aquiluna* MAGs through phylogenomic and pangenomic analyses

**Fig 7:**
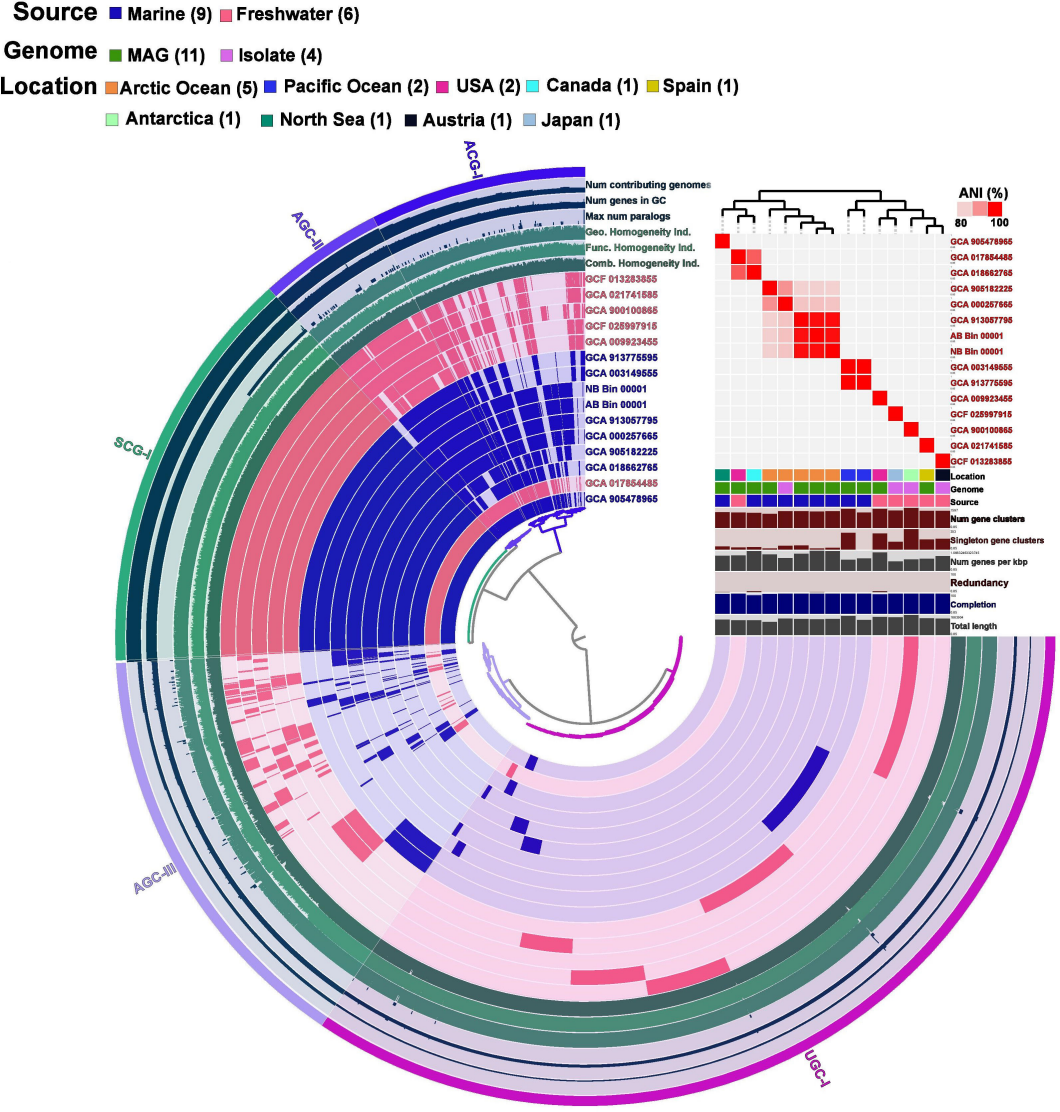
Pangenomic analysis of MAGs (*n* = 15) belonging to the genus of *Aquiluna*. The circle diagram was based on the presence/absence of 3,764 gene clusters (GCs), which are represented by each layer of a single genome. The different color codes in the legends are represented by the source, genome type, and location of the MAGs from where they are retrieved. The GCs are categorized based on their frequency among all the genomes and are represented as single-copy core gene clusters (SCG), accessory gene clusters (AGC), and unique gene clusters (UGC) of the genomes. The other outer rings represent various MAG properties among each genome, whereas average nucleotide identity (ANI) values are also represented as heatmaps.

In the present study, we have identified two potential novel MAGs (AB_Bin_00001, NB_Bin_00001) belonging to the genus *Aquiluna,* mainly found to have high coverage at AB and NB stations (Table S8), which was also abundant based on amplicon sequencing data (*Microbacteriaceae* family) in AB and NB (Fig. 3B). However, their potential ecological roles have not been studied in detail. Interestingly, all the MAGs that are publicly available and used for comparative analysis in the present study belonged to marine and freshwater ecosystems (Table S12). The phylogenomic analysis revealed that MAGs and isolate genomes within this group were composed of three separate clusters (marine clusters I and III and freshwater cluster II). The two reconstructed MAGs from the current study were found to be phylogenetically closely associated with the MAGs (GCA_000257665, GCA_905182225, GCA_913057795) retrieved from the Arctic Ocean with the ANI percentage ranging from 76% to 92% (Fig. S6; Table S13). These MAGs were phylogenetically very distinct from the isolate *Aquiluna boronia* (ANI: 29%), suggesting that these MAGs potentially belong to novel bacterial species (Fig. S6). The pangenomic analysis revealed that all the MAGs (*n* = 15) belonging to the genus *Aquiluna* consist of 3,764 gene clusters (20,550 genes), of which about 18.7% of single-copy core genes (*n* = 707) were shared among them (Fig. 7). The analysis further revealed the distribution of accessory genes (i.e., genes present in more than one genome but not in all the genomes), which consist of about 35.3% (*n* = 1,327) of the total pangenome, whereas 46% of the genes (*n* = 1,730) were categorized as singletons (i.e., genes only present in a single genome) among the studied genomes. The SCGs of *Aquiluna* were found to have metabolic functions associated with amino acid utilization, fermentation, oxidative phosphorylation (complex II, V), and iron/manganese reduction, whereas AGCs were mainly responsible for oxidative phosphorylation (complex IV), C1 metabolism, and complex carbon degradation processes (Table S14). Meanwhile, the UGCs were found to have only a few functions associated with sulfide oxidation and complex carbon degradation processes.

## DISCUSSION

### Isotopic and back- trajectory data reveal distinct origins and ages of sea-ice floes in the CAO

The back-trajectories indicate that the three sampled sea-ice floes originated from geographically distinct regions (Fig. 1B). The ice at the NP and AB stations showed distinctly lower isotopic values, indicative of growth into lower-salinity water, pointing to an origin further east, also indicated by the back-trajectories, affected by low-salinity surface shelf waters entering the Arctic basin. While the ice at the NP station was relatively thick, and the salinity and morphology of the ice floe suggest this being thick FYI. The very desalinated ice at the AB station, however, points to this being older [second-year ice (SYI) or older MYI], having experienced melt over at least one summer season. The ice in the NB was sourced from Laptev Sea in the Eurasian Basin, and the sea-ice back-trajectory length suggests the oldest ice in the area could be SYI. However, the higher stable isotopic values indicate growth into seawater of higher salinity found in the eastern Eurasian Basin off the shelf, and the relatively high salinity of the ice itself indicates the sampled ice was most likely FYI.

### Contrasting community structure and patterns that are mainly shaped by ice floe age, origin, and localized environmental conditions

This study identified distinct bacterial community patterns across three Arctic ice floes classified as FYI (NP and NB) and SYI/MYI (AB). Analysis of targeted 16S rRNA gene amplicon sequencing showed a demarcation of bacterial community assemblages according to ice horizon, highlighting the vertical structuring of microbial communities within the sea ice (20, 23–32, 35–37). However, we could not apply the MAG-based genome-resolved metagenomics approach among different ice horizons because of a paucity of DNA concentrations in the samples. Despite this limitation, we were able to reconstruct MAGs of the majority of the dominant bacterial taxa reported by targeted sequencing (Table S3), thus enabling insights into their genomic features, functional capabilities, and ecological roles within Arctic sea-ice habitats. Targeted amplicon analysis showed bacterial taxa *P. psychrophila*, *O. arcticus,* and *Polaribacter irgensii* dominance in MYI (AB) bottom horizons, while *A. antarcticum, O. antarcticus, Polaromonas cryoconiti, Rhodoferax* sp., *N. dokdonensis,* and *R. ferrireducens* were observed in the bottom horizons of FYI at NP and NB (Fig. 3; Table S3).

The repeated freezing and melting events over successive years create strong compartmentalization across MYI horizons that allow bacterial communities to adapt and colonize, whereas FYI in polar oceans forms annually and is influenced by immediate oceanic, atmospheric, and riverine inoculations, resulting in a higher prevalence of diverse bacterial taxa (19, 20, 24, 27, 36). This was also further evidenced in our observation that the FYI (in NB) contains the highest number of unique species (*n* = 203), which may be reflected by localized hydrographic conditions like the influence of Atlantic water inflow in the region, leading to distinct microbial assemblages (20, 37). Besides, the bacterial community composition across the horizons of the MYI cores at the AB ice station was also found to be different and fairly consistent with the previous literature. Similarly, relative abundance profiles of certain bacterial taxa inhabiting the top and middle cores differed notably between FYI and MYI (e.g., *F. frigoris and Hydrogenophaga* sp.). Additionally, specific bacterial taxa, such as *Polaromonas cryoconiti, R. ferrireducens, and Rhodoferax* sp. Gr-4, etc., exhibited pronounced differences between the two FYI ice stations (NP and NB), suggesting that localized environmental drivers also play a crucial role in shaping community assemblages within the sea-ice habitat (24, 25). Our observations indicate that despite low concentrations of NO_3_ and NO_2_ at the NP ice station, it showed a correlation with heterotrophic bacterial species like *F. frigoris and L. salsilacus*, suggesting that these groups promote the utilization of available dissolved organic substrates in the sea-ice matrix for their cellular growth and other metabolic processes (6, 38, 39).

The back-trajectory data suggest that the ice pack found at NP was traced back to the northern Chukchi Sea, an area well-known for receiving a substantial influx of nutrient-rich Pacific water through the Bering Strait (40), which likely contributed to the unique community diversity at the NP ice station. Similarly, the ice pack in at AB was traced back to the East Siberian Sea, an area well known for receiving larger amounts of nutrient-rich freshwater containing terrestrial-derived organic matter from the surrounding Lena and Kolyma rivers (41, 42). The ice-core composition also indicates that the AB ice station was relatively less saline and inhabited by a specialized group of bacteria like *Paraglaciecola psychrophila*, *G. pallidula* (*Alteromonadaceae), Rhodoluna lacicola*, *R. limnophila,* and Ca. *Aquiluna rubra* (*Microbacteriaceae),* which is typically found in environments such as sea-ice melt ponds and freshwater habitats like rivers and lakes (7, 43). However, some of these bacterial species were also found in NB (which had more melt ponds at the time of sampling), likely indicating that localized habitats like melt ponds might have played a crucial role in shaping these groups. Similarly, the dominant bacterial species (e.g., *N. dokdonensis). O. arcticus* and *Polaribacter irgensii*) found in NB, along with their relationship with δ^18^O and SiO₄, suggest that this ice floe likely originated in diatom-rich saline waters, where these bacterial groups may have established symbiotic relationships with ice algae like *Melosira arctica* (15, 24, 25, 35).

### Prevalence of sulfur cycling and complex carbon degradation pathways in the sea ice

Nearly all the MAGs (21 of 25) belonging to diverse bacterial genera like *Flavobacterium, Rhodoferax, Aquiluna, Algoriphagus, Polaromonas, Hydrogenophaga, RS62, Nonlabens, Unclassified_Cellvibrionaceae, Octadecabacter, Polaribacter, Paraglaciecola, and NORP294* were found to be encoded with dimethyl-sulfide monooxygenase (*dmoB*). The prevalence of metabolic gene encoding for *dmoB* at all three stations suggests that microorganisms transform volatile dimethyl sulfide (DMS) into a more stable dimethyl sulfoxide (DMSO) sulfur compound, which is being further recycled by microorganisms for their energy production and cellular growth (44–46). DMS is a by-product of DMSP, which is mainly produced by ice algae (5, 18, 24, 45–47) that abundantly thrive during the summer ice melt season. Likewise, metabolic genes encoding for dissimilatory sulfur metabolism, mainly sulfur oxidation (*sdo*), were also commonly observed in metagenome assemblies and MAGs (15 of 25) at all ice stations, suggesting that sulfur oxidation is widespread in sea-ice microbial communities and contributes significantly to sulfur recycling in sea ice (5, 48). A recent metagenomic study on FYI prokaryotic communities associated with ice algal blooms in Hudson Bay, Canada, was found to possess genes for DMSP degradation and sulfur oxidation processes among heterotrophic bacterial groups associated with Gammaproteobacteria and Campylobacteria (5). Our observations of distinct taxonomic diversity between FYI and MYI among three stations and the prevalent identification of sulfur-related pathways at all ice stations suggest the presence of high metabolic functional redundancy among the microorganisms (49, 50).

Bacterial communities inhabiting such sulfur-rich environments might have acquired sulfur-associated genes through horizontal gene transfer mechanisms from those specialized sulfur-oxidizing bacterial groups for their energy requirements, survival, and efficient recycling of sulfur compounds in sea-ice habitats (49, 50). Interestingly, we have also observed metabolic genes encoding for thiosulfate oxidation (*sox A, B, C, X, Y, and Z*) exclusively in the AB and NB ice stations. Thiosulfate is an intermediate product mainly produced from the decomposition of organic matter. One of the possible likelihoods for these observations is that the ice pack found in AB and NB was traced back to the source of origin in the Siberian seas, which generally receive higher terrestrial DOM content from the surrounding rivers (41, 42). The complex structures of these DOM molecules likely persist in sea ice for an extended period, requiring more time for microbial breakdown. This was further evidenced by the prevalence of metabolic genes associated with complex carbon degradation, such as cellulose degrading (beta-galactosidase), chitin degrading (hexosaminidase), xylan degrading (beta-xylosidase), amino acid utilization (*pabC*), and fatty acid degradation (*hadH*), at the AB and NB stations in comparison to NP (Fig. S5). The heterotrophic bacterial groups inhabiting the AB and NB stations are more effective at recycling carbon through the degradation of complex organic compounds found in sea ice. These complex carbon degradation-associated metabolic genes were found to be encoded in the MAGs belonging to *Flavobacterium* (NP_Bin_00001, NP_Bin_00004, AB_Bin_00002, AB_Bin_00003, AB_Bin_00006, NB_Bin_00005), *Rhodoferax* (NP_Bin_00002), *Polaromonas* (NP_Bin_00003, AB_Bin_00005), *Hydrogenophaga* (AB_Bin_00008), *RS62* (AB_Bin_00009), *Paraglaciecola* (AB_Bin_00012, NB_Bin_00008), *Cellvibrionaceae* (NB_Bin_00002), *Nonlabens* (NB_Bin_00003), *Octadecabacter* (NB_Bin_00004), and *Polaribacter* (NB_Bin_00006). Targeted amplicon sequencing analysis also revealed that the majority of these bacterial genera were highly abundant across the ice horizons in both FYI and MYI (Fig. S3B), indicating that these carbon degradation processes are prevalent in sea ice. During the summer ice melt season, algal biomass-derived complex organic matter is usually rich in abundance and is usually decomposed by these heterotrophic bacterial groups, thereby contributing to carbon recycling within the sea-ice habitat (4, 21, 51). During such carbon degradation processes, some of these bacterial groups also influence nutrient dynamics, especially the removal of bioavailable nitrogen from the system (52).

The observation of metabolic genes encoded for denitrification processes (*nirB, nirD, and nirK*) among the three sea-ice stations suggests that these metabolic processes are particularly found in environments where oxygen is limited or absent, such as the hypoxic zones within sea-ice brine channels due to heterotrophic bacterial activity (5, 53). Such anoxic conditions were previously reported in Antarctic winter sea ice containing dense algal accumulations with fairly consistent dominant bacterial genera reported in the current study (54). Similarly, S. Rysgaard et al. (55) have reported high denitrification activity from the bottom sections of Arctic sea ice during the summer sea-ice melt season. Observations of these denitrification pathways were particularly identified in the MAGs (Fig. 6) belonging to bacterial genera like *Rhodoferax* (NP_Bin_00002), *Polaromonas* (NP_Bin_00003), *Hydrogenophaga* (AB_Bin_00008), and *Paraglaciecola* (NB_Bin_00008), suggesting that these bacterial groups play a crucial role in regulating nutrient availability by adapting to fluctuating oxygen levels during algal bloom conditions and thus impact the overall primary productivity (5). Overall, the metabolic functional analysis suggests that functional redundancy is commonly found in pathways associated with sulfur cycling and degradation of complex organic matter among the distinct microbial groups in sea-ice habitats, where multiple species perform similar ecological roles, contributing to the stability of ecosystems.

### Small genome with big influence: key survival pathways of *Aquiluna* and its ecological role in the environment

Based on the ANI data, it is evident that two reconstructed *Aquiluna* MAGs potentially belong to novel species, and their ecological functions in the sea-ice habitat and other environments are not well known. Their genome size ranges from 1.3 to 1.6 Mb and is considered a free-living chemo-organotrophic bacterial group (56, 57). The comparative genomic analysis of *Aquiluna* MAGs showed high genomic diversity within the group with a unique metabolic repertoire of genes that encode for different metabolic functions (Table S14). The reconstructed MAGs were closely associated with the marine cluster I clade, which mainly comprised MAG data sets retrieved from the Arctic Ocean samples; however, they were phylogenetically distinct from the isolate genome *Aquiluna boronia* (Fig. S6). The SCGs were mainly found to be responsible for generating ATP through oxidative phosphorylation processes (Complex II & IV), the energy needed to power cellular functions in the bacterium to survive extremely harsh climatic conditions such as sea-ice habitats (39, 58). The AGCs were found to be mainly responsible for the degradation of different complex organic matter such as cellulose, xylan, starch, glycogen, pullulan, and amylopectin (Table S14) derived from plant cell and algal origin (5, 59, 60). Thus, the genus *Aquiluna* critically contributes to carbon cycling and nutrient recycling, thereby maintaining the ecological balance in the sea-ice habitats. Similarly, all the analyzed MAGs among the *Aquiluna* were also found to encode for the metal reduction processes (iron/manganese). This process plays a critical role in relatively low oxygen or oxygen-depleted zones like sea-ice brine channels, the taxa belonging to *Aquiluna* can use metals like iron and manganese in their higher oxidation states as electron acceptors to facilitate the breakdown of complex organic matter (39, 61). The identification of *Aquiluna MAGs* only in the AB and NB suggests that they play a critical role in the degradation of different complex organic matter (see earlier discussion) present in the sea ice.

### Conclusions

Here, we provide key insights into the bacterial community structure inhabiting different ice horizons of FYI and SYI/MYI, including species-level identification and their metabolic functions in sea-ice floes sourced from different regions of the Arctic Ocean. The ice floe age, origin, and localized physicochemical conditions of the ice together shaped the distinct bacterial assemblages observed in the sea-ice habitat. We also report the presence of key bacterial species belonging to Bacteroidota, Proteobacteria, and Actinobacteriota and their potential source of origin linked to interactions with freshwater inputs from Siberian rivers. Further, we also demonstrated functional redundancy by microbial communities in the sea-ice habitats with their critical roles associated with sulfur and complex carbon degradation processes. We have also deciphered the potential ecological role of novel MAGs belonging to *Aquiluna* with complex organic matter degradation. With continued sea-ice reduction (10) and shift to predominantly FYI, these microbial processes may be significantly altered, affecting the broader Arctic marine ecosystem. Overall, this research underscores the need for continued monitoring of microbial community shifts in response to rapid Arctic environmental changes.

## MATERIALS AND METHODS

### Collection of sea-ice core samples

For genomic analysis, two sea-ice cores (biological replicates) from three ice stations, namely, at the North Pole (NP), in the Western Amundsen Basin (AB), and the Western Nansen Basin (NB), were collected during the Norwegian Polar Institute’s Arctic Ocean cruise (AO2022) on board FF *Kronprins Haakon* during the period between July and August 2022 (Table S1; Fig. 1). At each site (ice floe), a location on the floe that seemed representative of the level ice was chosen to collect the ice cores. Representativeness of the coring sites was further assessed by local (ice floe) -scale ice and snow thickness surveys using the method of walking transects with the electromagnetic instrument GEM-2 and snow probe (see P. Itkin et al. (62) for details on the methodology). At the selected coring sites, the ice thickness was about 1.9 m at the NP ice station, whereas in the AB, it ranged from 1.8 to 1.9 m, and in NB, it was about 1.3 m. The snow layers on the sea-ice surface were removed, and the ice cores were recovered using a 9 cm diameter Mark II coring system (Kovacs Enterprise LLC, USA). Before and after each ice station, the coring equipment was cleaned thoroughly by rinsing with purified water. Albeit during each coring event, the corer is also unavoidably rinsed with local seawater when the cores are extracted. The collected ice cores were divided into three equal sections aseptically (Top, Middle, and Bottom) based on the total length of the ice cores and melted in darkness in sterile polyethylene bags (Whirl-Pak) at 4°C for about 48–60 hrs without the addition of filtered seawater to avoid possible external DNA or nutrient contamination of the samples (54, 63, 64). The resulting meltwater for each sample horizon (about 2 L) among three ice stations was filtered at room temperature for about ~30 mins through 0.22 µm polycarbonate membrane filters (Merck-Millipore) to retain microbial biomass. The filters were immersed in RNAlater (Invitrogen) and stored at −20°C until further analysis.

### Physical and chemical properties of ice cores

For the analyses of dissolved nutrients, the abovementioned melted sea-ice core samples were collected into acid-washed 60 mL polypropylene tubes and immediately stored frozen until analysis. Nitrite (NO_2_), nitrate (NO_3_), phosphate (PO_4_), and silicate (SiO_4_) concentrations were determined using a SmartChem200 discrete analyzer (AMS Alliance). An additional core was also collected for the analysis of the physical properties of sea-ice. This follows the methods outlined in Divine et al. (65). Sea-ice temperature profiles (core 1) were measured using a Testo 720 (Testo SE & Co. KGaA, Germany) thermistor probe, into holes drilled at 5 or 10  cm distance, typically within couple minutes of core extraction to ensure a minimal alteration to the initial temperature profile of the ice slab. The vertical profiles of sea-ice salinity (on the practical salinity scale, unitless) and stable water isotopes (δ^18^O, reported in per mil (‰) against V-SMOW) were acquired from a core that was cut on-site in 5 cm long sections immediately after recovery and brought onboard in sealed containers for melting. The bulk salinity of melted ice sections was then measured using a conductivity meter Cond3110 SET3 (practical salinity, unitless). Melted ice samples were further subsampled for stable oxygen isotopes (δ^18^O reported in per mil (‰) against V-SMOW) and filled into 20 mL scintillation vials that were closed tightly (and sealed with parafilm) until analysis at the Norwegian National Infrastructure project FARLAB (Facility for advanced isotopic research and monitoring of weather, climate, and biogeochemical cycling) at the University of Bergen (Norway). At FARLAB, samples were transferred to 1.5 mL glass vials. An autosampler (A0325, Picarro Inc, USA) transferred ca. 2 uL per injection into a high-precision vaporizer (A0211, Picarro Inc, USA) heated to 110°C. After blending with dry N_2_ (< 5 ppm H_2_O), the gas mixture was directed into the measurement cavity of a Cavity-Ring Down Spectrometer (L2140-i, Picarro Inc, USA). Two or three standards were measured at the beginning of each run, which consisted typically of 20 samples, with a drift standard measured typically every five samples. Internal reproducibility was estimated from the 1-sigma standard deviation of repeated measurements of a drift standard to 0.066‰.

### Calculation of sea-ice back-trajectories

The on-ice location was tracked in a Lagrangian manner by calculating each daily position according to the sea-ice drift for that day resampled to that position. The method is similar to that of Kimura et al. (66) but tracking backward in time instead of forwards. The trajectory ends where the condition of an ice concentration below 15% is met for three consecutive timesteps. This provides a range of the most likely endpoints, both in time and space, of the back-trajectories of the area of ice floes on which the sea-ice stations were located. All sea-ice data used in the backtracking are from the EUMETSAT Ocean and Sea Ice Satellite Application Facility (OSI SAF, https://osi-saf.eumetsat.int/products/osi-450-a1). The sea-ice drift data were derived from multiple passive microwave sensors. The sea-ice drift climate data record version 1 (OSI SAF, 2022a) (67) was used for dates up to and including 2020 and the sea-ice drift near real-time product (OSI SAF, 2010) for dates from 2021 onward (68). The sea-ice concentration was also based on selected passive microwave sensors. The sea-ice concentration climate data record version 3 (OSI SAF, 2022b) was used for dates up to and including 2020 and the accompanying interim climate data record version 3 (OSI SAF, 2022c) for dates from 2021 onward (69, 70). The sea-ice drift products used in calculations have a spatial resolution of 75 km (climate data record) and 62.5 km (near real-time product). The trajectories contribute to an overall picture of sea-ice drift towards the ice station locations on a larger scale, and they cannot be considered a set of precise drift trajectories for individual ice floes or their exact origin or age. In essence, the trajectories indicate the maximum possible sea-ice age in the region of an ice station.

### Genomic DNA extraction, metabarcoding, and metagenome sequencing of samples

The genomic DNA was extracted from a total of 18 microbial biomass membrane filters that were collected from three ice stations using the DNeasy Powersoil Pro kit (Qiagen, USA). The concentration of extracted genomic DNA was assessed using the Qubit dsDNA HS Assay Kit (Thermo Fisher, USA). The DNA concentrations varied from 6 to 21 ng/µL for the extracted samples. About 10 ng of genomic DNA was used to amplify the full length of the 16S rRNA gene by PCR using a rapid 16S barcoding kit (SQK-16S024) from Oxford Nanopore Technologies (ONT, UK) according to the manufacturer’s protocol (71, 72). A negative control reaction was also performed during the PCR to ensure that the reagents were free from contamination. The barcoded amplicon libraries were purified by AMPure XP magnetic beads (Beckman Coulter, USA) and quantified using the Qubit dsDNA HS Assay Kit (Thermo Fisher, USA). The barcoded amplicon sequence libraries were pooled in equal molar concentrations and loaded on the R.9.4.1 (FLO-MIN106) flow cell (ONT, UK) as per the recommended protocol. The sequencing was carried out in the MinION Mk1B device (ONT, UK) using the MinKNOW v23.11.5 software for up to 72 hours. The median read length obtained among the samples varied from 1,249 bp to 1,529 bp, while the majority of the samples were found to have about 1,449 bp. Similarly, for metabolic functional analysis, genomic DNA was pooled from all the samples (Top, Middle, and Bottom) collected from each ice station to obtain sufficient required concentration for the library preparation. The three metagenomic libraries representing three ice stations were prepared using the SQK-LSK110 ligation sequencing kit (ONT, UK). The libraries were then loaded onto R.9.4.1 nanopore flow cells and sequenced on the MinION Mk1B device (ONT, UK) as described above. The generated sequence data sets were also submitted to the NCBI sequence read archive portal under bio project ID: PRJNA1172671.

### Bioinformatics analysis of amplicon and metagenomic sequence data

The raw nanopore amplicon (16S rRNA gene) and metagenomic sequence data sets were base-called using the Guppy v.6.5.7 software using the “dna_r9.4.1_450bps_sup.cfg” model. The amplicon data analysis for species-level microbial community profiling using full-length 16S rRNA sequence data sets was carried out by Emu v.3.4.5 (72) with a custom-curated Emu database developed from Silva reference database V138.1. Visualization of amplicon data was carried out using the “microeco” R package v1.13.0 (73). Sequence reads that belonged to chloroplast, mitochondria, and sea-ice algae were removed during downstream analysis before plotting graphs. The NMDS analysis of amplicon data was carried out by using the multivariate statistical software Primer v7 (PRIMER E) (74). The RDA analysis was carried out to investigate the influence of environmental parameters on bacterial community structure using Canoco v5 (75). To identify statistically significant and differentially abundant bacterial taxa across ice horizons and ice stations, a linear discriminant effect size (LEfSe) analysis was carried out (76). The metagenomic sequence data sets were assembled into contigs by the *de novo* assembly approach by using three different assembly software, namely, NanoPhase v.2.0.3 (77), MetaBooster (78), and metaFlye v.2.9.3 (79). The metagenome assemblies were further quality-checked using MetaQUAST v.5.0.2 (80), and the best optimal assembly obtained from NanoPhase was further used for downstream analysis. The metagenome-assembled genomes (MAGs) were reconstructed from the metagenome assemblies using Anvio v7.1 (81). The MAGs were reconstructed by reference-based assembly workflow (https://merenlab.org/2016/06/22/anvio-tutorial-v2/) with the “all against all” parameter in Anvio v7.1 (81, 82). The major steps include the identification of open reading frames using Prodigal v2.6.3 (83), and the annotation of single-copy core genes was done by HMMER v3.2.1 (84). The sequence coverage of each assembly was calculated by using bowtie2 v2.3.5.1 (85). Then, the anvi-profile program was used to generate coverage and detection statistics of metagenome assemblies. The mean coverage value of each MAG was obtained by summing the coverage of each nucleotide in a contig and dividing by the length of the contig. The automated binning was performed on assemblies using three separate binning tools, namely, CONCOCT v1.1.0 (86), maxbin2 v2.2.7 (87), and METABAT2 v2.12.1 (88) to generate MAGs. The anvi-refine program was used interactively to refine the MAGs that contain high redundancy. Finally, DAS_Tool v1.1.2 (89) was used to dereplicate and select the MAGs that contain completeness of >50% and redundancy of <10%. The taxonomic identification of MAGs was carried out by using the GTDB-Tk tool v1.5.1 against reference database V207 (90). A total of 236,976 genes were annotated via Prodigal with an average of 78,992 genes per sample among the three sea-ice stations. Both metagenome assemblies and MAGs were further subjected to functional profiling using METABOLIC v4.0 (91) to identify the pathways associated with carbon, nitrogen, and sulfur cycles. The identified metabolic gene counts (from Hidden Markov Model; HMM_hit) for each metabolic function/pathway associated with the above-mentioned processes were normalized by the Z score and plotted as a heatmap using “complexheatmap” R package v2.25.2 (92). The reconstructed MAGs were submitted to the public domain and can be downloaded using the following link: https://figshare.com/s/16e8bf968df559d3322b.

### Phylogenomic and pangenomic analysis of novel *Aquiluna* MAGs

In the present study, we reconstructed MAGs belonging to the genus *Aquiluna*. To date, the genus *Aquiluna* contains only one culturable representative, namely, *Aquiluna borgnonia*, which has been isolated from the freshwater ecosystem (57). To identify the potential ecological roles of this particular genus, publicly available MAG data sets (*n* = 20) have been downloaded from the NCBI database, of which only a high-quality nonredundant set of MAGs (*n* = 13), along with MAGs from the present study, were used for further analysis. The phylogenomic analysis was carried out by the PhyloPhlAn v.3.0 package (93) with the following parameters (“-diversity high,” “-d phylophlan,” and “--accurate”). The generated phylogenetic tree was visualized using Anvio v7.1 (81). The pangenomic analysis was carried out as per the pangenomics workflow (https://merenlab.org/2016/11/08/pangenomics-v2/) using Anvio v7.1. The metabolic potential of the MAGs was identified using the METABOLIC v4.0 program (91). All the associated steps involved in the phylogenomic and pangenomic workflow were described in detail elsewhere (94).

## Data Availability

The sequence datasetsdata sets generated in this study are publicly available under the following NCBI Bio project ID: PRJNA1172671. The reconstructed metagenome assembled genomes from the metagenomic datasetdata set and all the bioinformatics scripts used in the current study were submitted to the public domain and can be downloaded using the following link: https://figshare.com/s/16e8bf968df559d3322b.

## SUPPLEMENTAL MATERIAL

Supplemental figures spectrum.01291-25-s0001.docx Figures S1 to S6.

Supplemental tables spectrum.01291-25-s0002.xlsx Tables S1 to S14.

ASM does not own the copyrights to Supplemental Material that may be linked to, or accessed through, an article. The authors have granted ASM a non-exclusive, world-wide license to publish the Supplemental Material files. Please contact the corresponding author directly for reuse.
