# Supplementary material for: Distinct bacterial community structures with abundant carbon degradation and sulfur metabolisms found in different sea-ice types from the Central Arctic Ocean
Source: Microbiol Spectr. 2025 Oct 8;13(11):e01291-25. doi: 10.1128/spectrum.01291-25 (PMC12584769; doi:10.1128/spectrum.01291-25)
Supplement: Supplemental figures — Figures S1 to S6. [file spectrum.01291-25-s0001.docx]

**Supplementary figures**

**Distinct bacterial community structures with abundant carbon degradation and sulfur metabolisms found in different sea ice types from the Central Arctic Ocean**

**Authors:**

Siddarthan Venkatachalam^1^*, Mats A. Granskog^2^, Rafael Gonçalves‐Araujo^3^, Dmitry V. Divine^2^, Puthiya Veettil Vipindas^1^, Thajudeen Jabir^1^, Ahammed Shereef^1^, Anand Jain^1^

**Affiliations:**

^1^Arctic Ecology and Biogeochemistry Section, National Centre for Polar and Ocean Research, Ministry of Earth Sciences (Govt. of India), Vasco-da-Gama, Goa, India.

^2^Norwegian Polar Institute, Fram Centre, Tromsø, Norway.

^3^National Institute for Aquatic Resources, Technical University of Denmark, Lyngby, Denmark.

**^*^Corresponding author:** Siddarthan Venkatachalam, Arctic Ecology and Biogeochemistry Section, National Centre for Polar and Ocean Research, Ministry of Earth Sciences (Govt. of India), Vasco-da-Gama, Goa, India.

Email: [venkatachalam@ncpor.res.in](mailto:venkatachalam@ncpor.res.in)


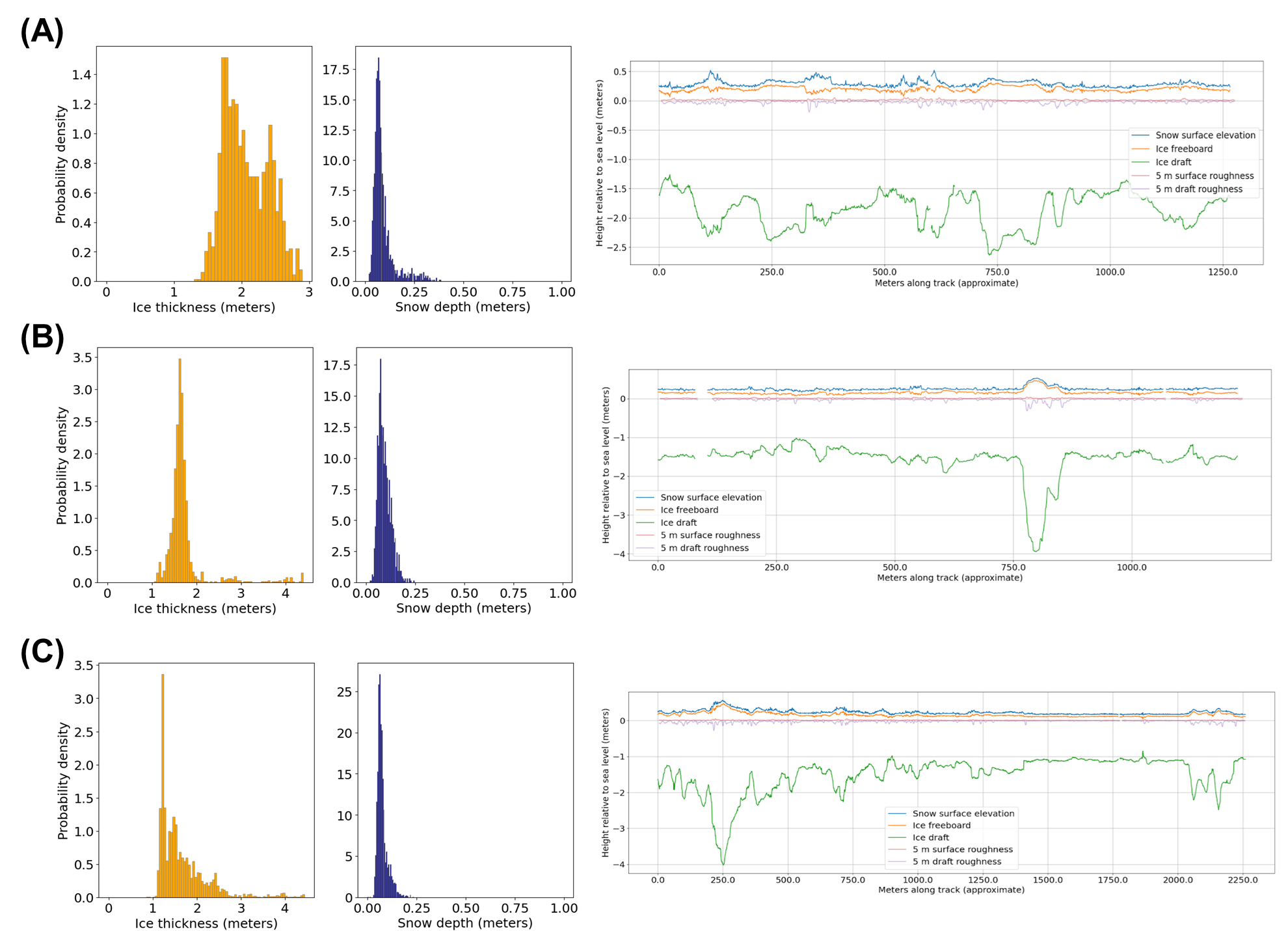


**Fig. S1.** The thickness of the sea-ice floe from the three sampling ice stations in the CAO region. (A) North pole, (B) Amundsen basin and (C) Nansen basin ice stations.


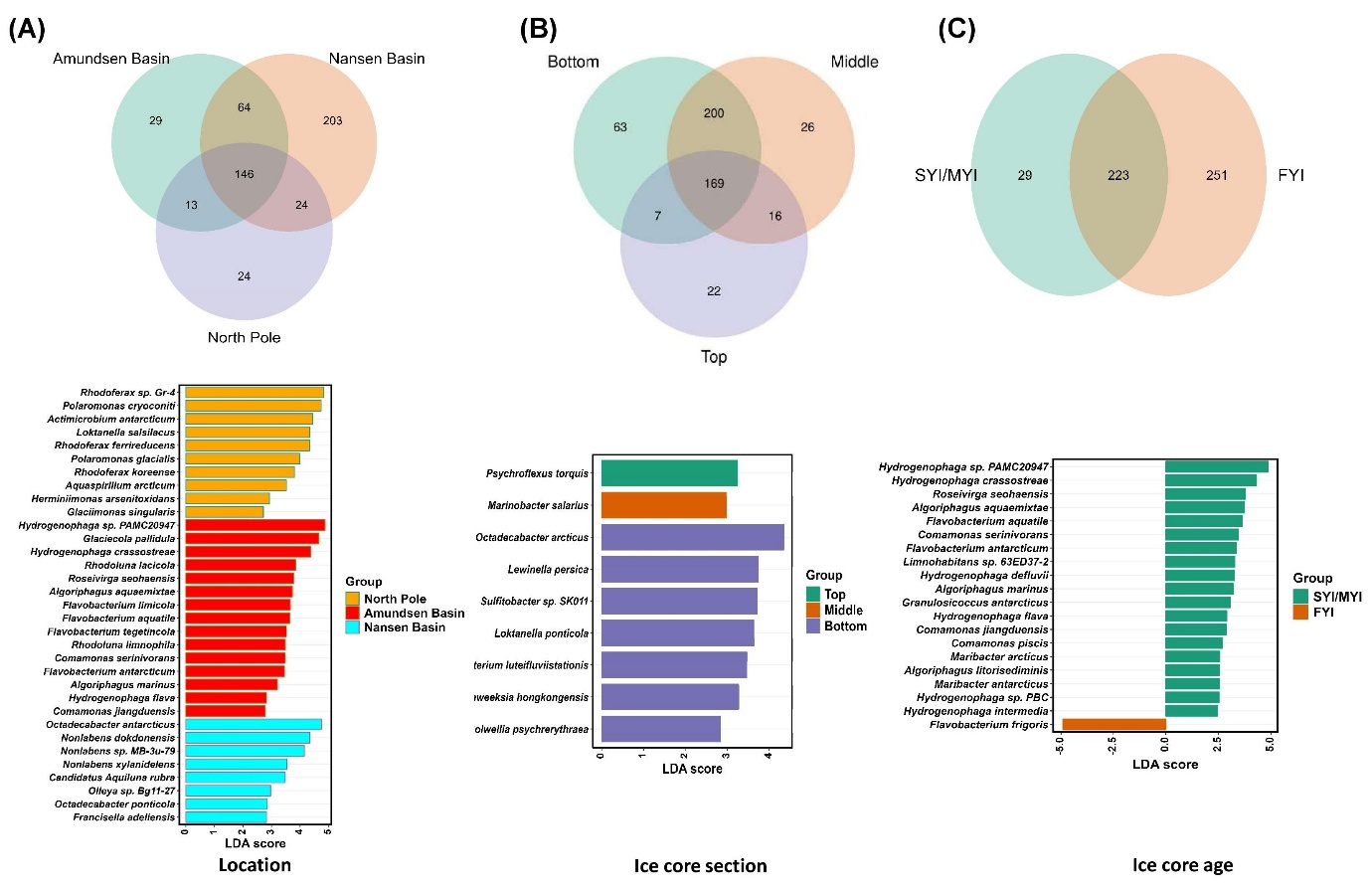


**Fig. S2.** List of unique, commonly shared and differentially abundant bacterial taxa among the three sea ice stations.


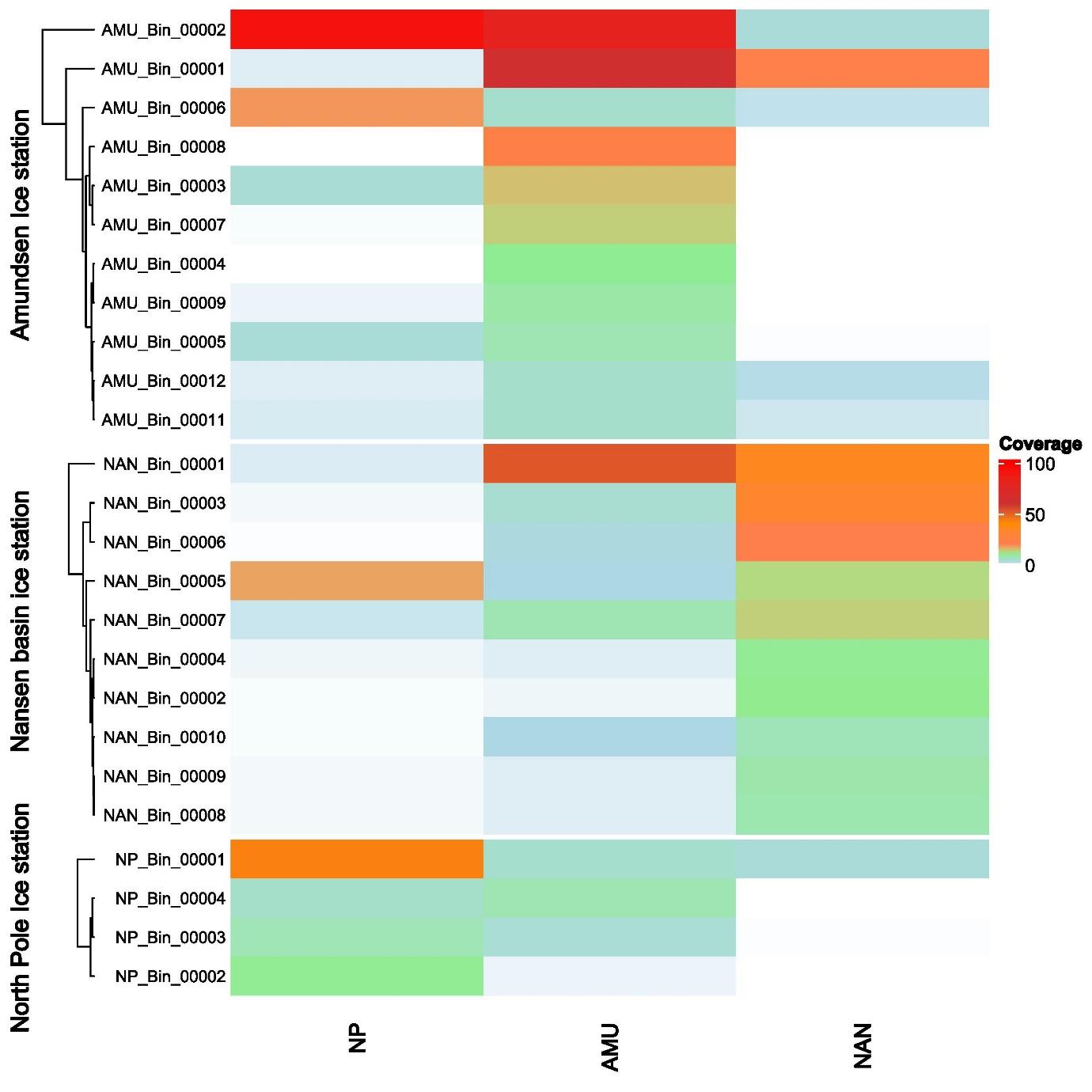


**Fig. S3.** The mean coverage of reconstructed MAGs among three Sea-ice stations in the CAO region. The mean coverage value of each MAG was obtained by summing the coverage of each nucleotide in a contig and dividing by the length of the contig.


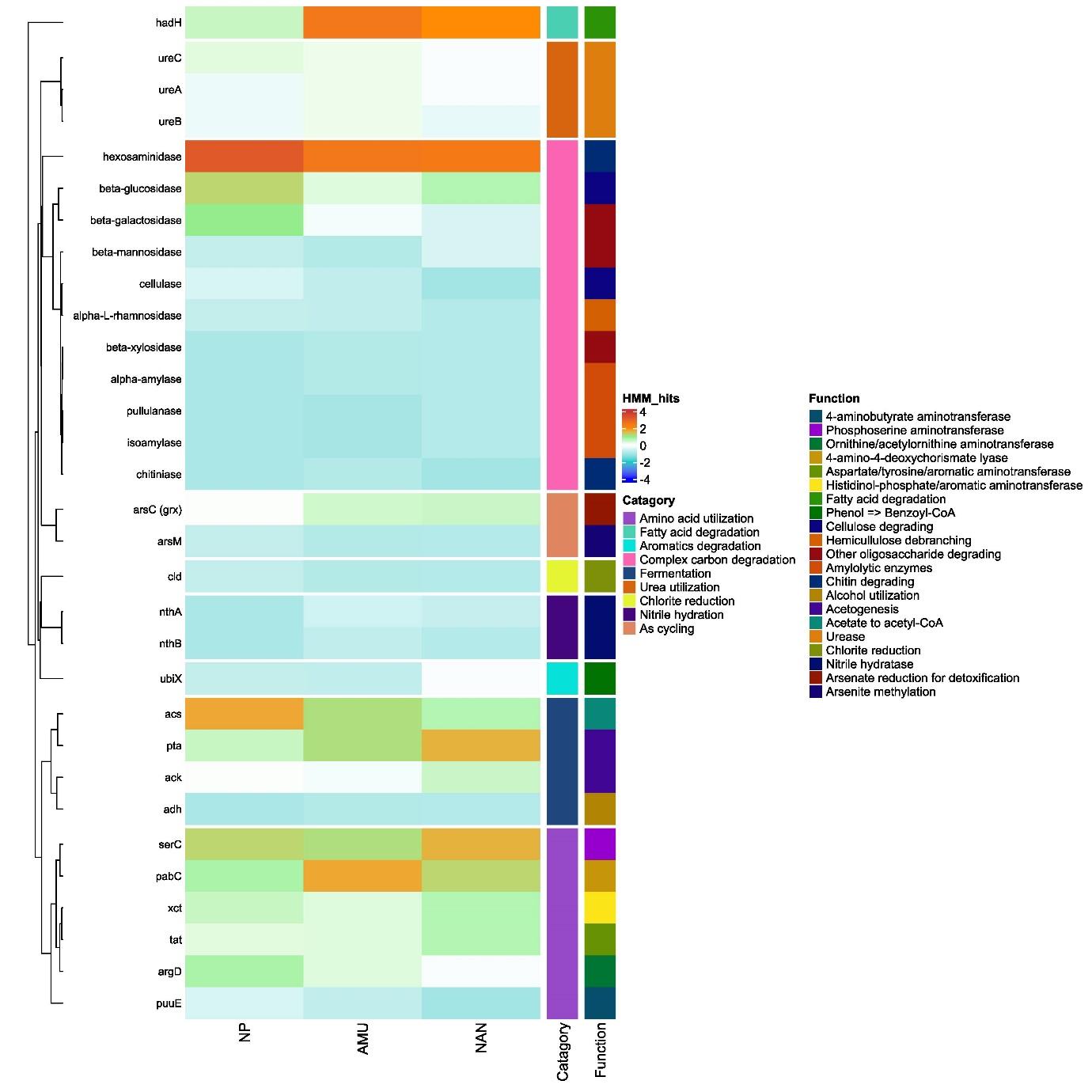


**Fig. S4.** Heatmap showing the various metabolic functions (other categories) found among the metagenome assemblies from all three ice stations. The metabolic gene counts (HMM_hit) of different processes were normalized using the Z score. A negative z-score (negative HMM_hit) indicates that the abundance of that particular metabolic gene in the sample is below the mean abundance across all samples, while a positive z-score indicates an above-average abundance. The bar plot color codes on the side represent the gene counts associated with various metabolisms and biogeochemical processes found within the reconstructed MAGs. The plot was generated using “complexheatmap” using the R package in R version 4.1.3.


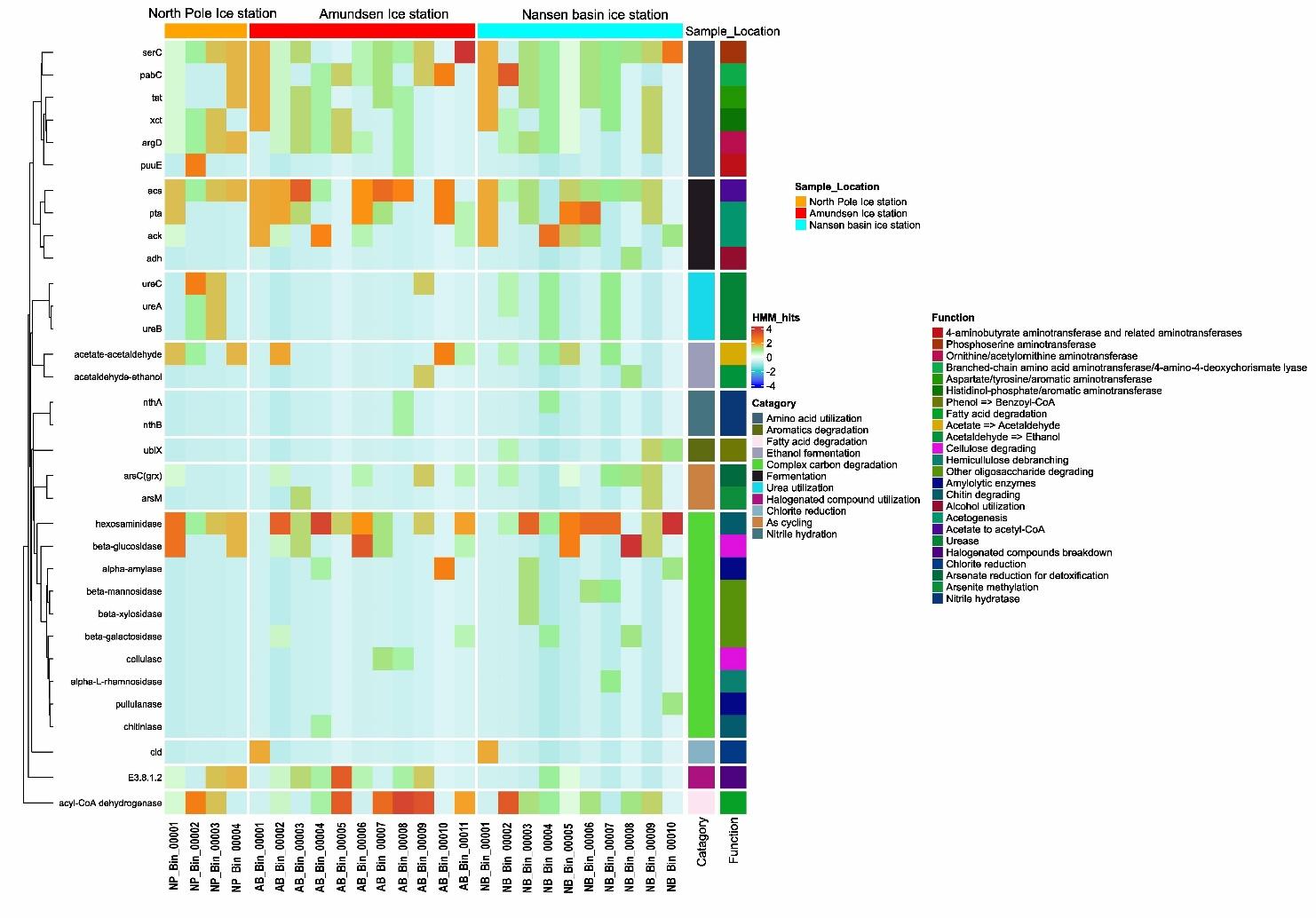


**Fig. S5.** Heatmap showing the various metabolic functions (other categories) found among the reconstructed MAGs from all three ice stations. The metabolic gene counts (HMM_hit) of different processes were normalized using the Z score. A negative z-score (negative HMM_hit) indicates that the abundance of that particular metabolic gene in the sample is below the mean abundance across all samples, while a positive z-score indicates an above-average abundance. The bar plot color codes on the side represent the gene counts associated with various metabolisms and biogeochemical processes found within the reconstructed MAGs. The plot was generated using “complexheatmap” using the R package in R version 4.1.3.


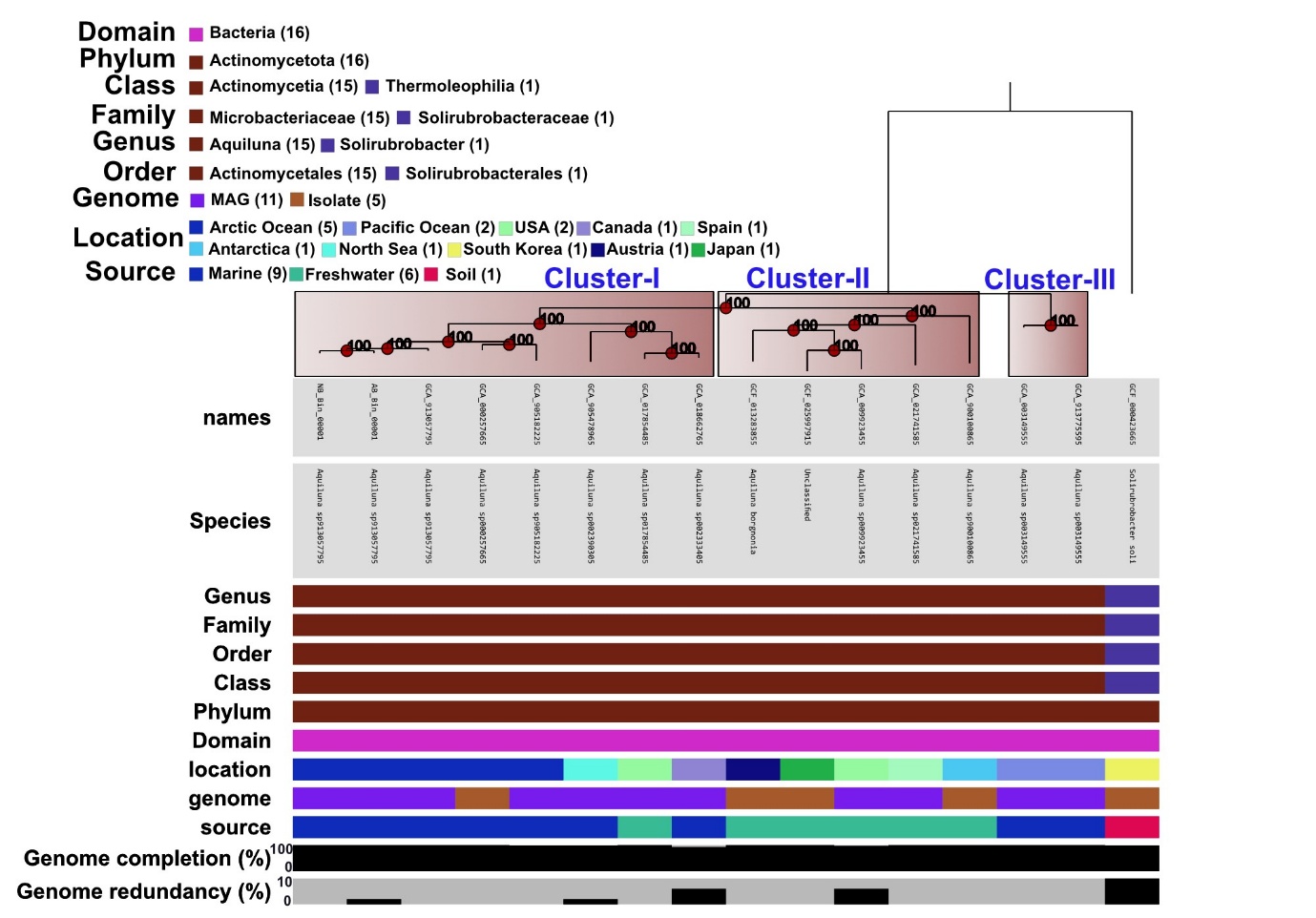


**Fig. S6.** Phylogenomic analysis of genus *Aquiluna* based on the publicly available isolate genomes and MAGs (n=15), which were recovered from diverse ecosystems. The bar graphs represent the taxonomic classification of MAGs at the Domain, Phylum, Class, Order, Family, and Genus levels, along with various MAG properties like Completion and Redundancy. The taxonomic description of each MAG, along with its source, location and genome detail, is color coded, and the description is provided in the figure (top left). The Isolate genome *Solirubrobacter soli* (GCF_000423665) was used as an outgroup. Bootstrap values are represented in the nodes of the tree.
